# Use of Tumescence for Outpatient Abdominoplasty and Other Concurrent Body Contouring Procedures: A Review of 65 Consecutive Patients

**Published:** 2015-09-01

**Authors:** Nathaniel L. Holzman, Mansher Singh, Stephanie A. Caterson, Elof Eriksson, Bohdan Pomahac

**Affiliations:** ^a^Langhorne Plastic & Reconstructive Surgery, Langhorne, Pa; ^b^Division of Plastic Surgery, Brigham & Women's Hospital, Boston, Mass

**Keywords:** abdominoplasty, outpatient procedure, tumescence, complications, pain

## Abstract

**Introduction:** Abdominoplasty is being increasingly performed as an outpatient procedure. The role of tumescent technique in decreasing postoperative pain and hospital stay has not been extensively studied. **Methods:** We reviewed 65 consecutive patients who underwent tumescent abdominoplasty over 20 months by a single surgeon. All the patients were followed up for at least 1 year. The outcomes were evaluated in terms of systemic complications such as deep vein thrombosis and pulmonary embolism and local complications such as seroma, wound infection, and skin necrosis. **Results:** Of the 65 patient records analyzed, 61 were of females and 4 of males. Average age for the patient population was 45.2 years. Mean follow-up was at least 1 year for all the patients. Ninety-five percent of patients could be discharged the same day with tumescent abdominoplasty, whereas 71% of the patients who underwent concurrent procedures with abdominoplasty were also able to go home the same day. All the patients reported excellent postoperative pain control. There was no report of deep vein thrombosis or pulmonary embolism in any of these patients. Wound complications occurred in 14 patients (21.6%), of which 12 patients had seroma (18.5%) and 2 had wound infection (3.1%). The seromas were treated with repeated aspirations or Jackson-Pratt drain placement, whereas the wound infections resolved with outpatient antibiotics. **Conclusions:** The safety and efficiency of outpatient abdominoplasty can be further facilitated by utilizing tumescence. Tumescence helps the patients be discharged sooner, usually the same day, mobilize sooner, and rely less on oral narcotics at home.

The general goal of abdominoplasty is to optimize the aesthetic appearance of the abdomen with a safe predictable operation.^1^^-^3 Most patients wish to regain a slender-looking torso with a flat abdomen. They also like to have a better defined, narrower waist line, with taut skin and less fat. The umbilicus should have a natural appearance and the scars should be acceptable.^4^^-^6 The surgical and anesthesia techniques should be safe and cost-efficient. Matarasso et al^1^ have classified the candidates for abdominoplasty into 4 types, with type 1 requiring only liposuction, type 2 a so-called mini abdominoplasty, type 3 a modified abdominoplasty, and type 4 a full abdominoplasty. The latter 3 are also performed in conjunction with liposuction. Several surgeons, particularly Lockwood,^7^^,^^8^ have stressed the importance of not only the vertical vector but also the horizontal vector when performing an abdominoplasty. Numerous different incisions have been proposed over the years for abdominoplasty, but most surgeons prefer a mainly horizontal low abdominal incision located in or close to “bikini line.”^9^^-^11 Complications related to abdominoplasty have been very well described in the literature.^12^^-^19 In general, it is a safe operation, with deep vein thrombosis (DVT) and pulmonary embolism (PE) being the only rare serious systemic complications.^19^^-^22 The effort to decrease the postoperative morbidity led to the introduction of postoperative continuous infusion pain pumps.^23^^-^26 With adequate postoperative pain control, the patients met discharge criteria sooner and most of them could be discharged as a day surgery patients. The ability to perform abdominoplasty as an outpatient procedure reduces the burden on health care and also improves patient satisfaction. In few studies, use of tumescence with abdominoplasty has resulted in minimal blood loss and almost no need for pain management in the early postoperative period, and the patients could be discharged home within 3 hours of the completion of the procedure.^27^^-^30 The goal of this study was to investigate if tumescence would facilitate abdominoplasty to be performed as an outpatient procedure.

## METHODS

Institute review board approval was obtained for chart review of the patients. A total of 65 patients underwent abdominoplasty performed by a single plastic surgeon during a 20-month period. All of the patients were classified as Matarasso type IV and underwent a full abdominoplasty. Follow-up was at least 1 year. The records of these patients were retrospectively reviewed by an independent observer. The data collected from the medical records included age, sex, medical history, medications, allergies, history of smoking, and surgical history. Information about concurrent procedures performed, admission for observation, and complications was also noted. Subsequent office treatments and surgical procedures were recorded as well. Recorded wound complications were infections, seromas, hematomas, and skin necrosis.

### Operative technique

Abdominoplasty was very similar for all these patients. The only variability occurred in the lateral part of the incision. The incisions could extend from the anterior to the posterior axillary line depending upon the amount of skin that needed to be excised. A total of 48 patients (74%) also had 1 or more concurrent procedures that were performed along with abdominoplasty (Table 1). All markings were performed preoperatively with the patient in a standing position. The patients were encouraged to bring their bikini bottom or favorite underwear in order to allow the markings of the wound in a position that would be concealed in this clothing. Prior to the start of the operation, a first-generation cephalosporin antibiotic was administered intravenously or substituted appropriately in patients with documented allergy. Thigh high pneumatic compression boots and a Foley catheter for monitoring of urine output were used in all cases. No systemic antithrombotic medications were given. Following induction of general anesthesia and surgical preparation and drape, the markings were remeasured and reoutlined. Subsequently, the abdominal wall was infiltrated with tumescent solution (500 mg of lidocaine and 1 mg of epinephrine were diluted in 1000 mL of regular saline). A total of (or a maximum of) 1 L of tumescent solution was used in each case. The level of infiltration was just above the fascia and was extended at least 3 cm beyond the area of dissection in all directions. In a small subgroup of patients, additional liposuction was done in the hip and inner thigh areas using tumescence. The lateral cutaneous femoral nerves were marked before making the incision. In the suprapubic area extending past the anterior superior iliac spine, the incision was beveled superiorly to preserve the suprapubic fat. Sharp dissection ensued and a cautery was used in bipolar mode only for hemostasis. The abdominal flap was elevated to the xiphoid process in the midline and approximately 3 cm above the costal arch in the mammary line. Subsequently, plication of the rectus fascia was done from the xiphoid process to the umbilicus and then from the pubic symphysis to the umbilicus. The suprapubic abdominal flap was lifted from the fascia to allow this plication. The suprapubic fat was later sutured back in the same position with absorbable sutures between the anterior rectus sheet and the Scarpa fascia. In the majority of the cases, optimal plication could be achieved in the midline, but in cases of asymmetry, due to scoliosis or fascial attenuation, additional lateral plication oriented obliquely, transversely, or horizontally was done to achieve the desired shape and symmetry. Subsequently, excess skin was removed. With the operating table flat, staples were placed between the inferior skin border and as high as possible on the superior skin. An additional 2 to 4 cm of skin was then marked to be removed depending upon the elasticity of the skin. After this, fat deep to the Scarpa fascia was removed from the remaining superior abdominal flap. In addition, an approximately 4-cm discoid body of fat was removed underneath the umbilicus. A 2-cm wide strip of half the depth of the midline fat was removed from the xiphoid down to umbilicus. With the skin edges stapled together, the location of the umbilicus was marked. A V-shaped incision was then made approximately 2 cm above the location of the base of the umbilicus. A superior wedge was then removed from the umbilicus attempting to leave an umbilicus of the same perimeter as the V-shaped inset. Subsequently, the incision was closed with a deep dermal layer of interrupted 3-0 monocryl sutures and a superficial running interdermal layer of 4-0 monocryl sutures. After placing the first deep layer of sutures, liposuction was done in the pubic as well as around the lateral corner of the incision as necessary. Fat was only approximated when there was a gap between the superior and inferior fat layers. One 7-mm Jackson-Pratt suction drain was placed transversely below the umbilicus and another transversely above the umbilicus and brought out through the incision approximately 2 cm from its end. Steri-Strips were used for the wound.

### Postoperative care

Postoperatively, the patients were transferred to the Day Surgery Recovery Room. Standard criteria were used for discharge, and they were also required to ambulate before discharge. They were provided prescription for pain medication and 1 week of antibiotics, usually cephalexin. They were also given sheets to record suction drain output and were instructed to report this to the surgeon's office every other day. When the suction drain output decreased to less than 20 mL per drain per day, they were seen in the office and the drains removed. After drain removal, they were seen 2 weeks, 4 months, and 12 months later. The scars were treated with silicone gel for up to 4 months postoperatively starting when the Steri-Strips were removed approximately 3 weeks after the operation.

## RESULTS

Of the 65 patient records analyzed, 61 were of females and 4 were of males. Average age for the patient population was 45.2 years, with an average body mass index of 29.2 (Table 1). Mean follow-up was at least 1 year for all the patients. None of the patients were identified as smokers or received a diagnosis of diabetes, and only 2 patients had documented asthma. Surgical history revealed that 21 patients had at least 1 cesarean delivery (32.3%), 14 patients underwent prior other gynecological surgical procedures (21.5%), 12 patients had prior abdominal surgical procedures (18.5%), and 3 patients had previous abdominoplasty procedures (4.6%).

Of the 65 abdominoplasties, only 17 (26.2%) were performed as a stand-alone procedure. The vast majority (73.8%) were performed in combination with other procedures (Table 2). The most common combined procedures included adjunct liposuction, umbilical hernia repair, blepharoplasty/face-lift/adjunct liposuction, mastopexy, mastopexy/adjunct liposuction, breast augmentation/mastopexy, and breast augmentation/mastopexy/adjunct liposuction. Additional liposuction was not performed on the raised superior abdominal flap at the time of abdominoplasty. The total number of patients admitted was 15, of which only 1 underwent abdominoplasty alone.

**Figure 1:**
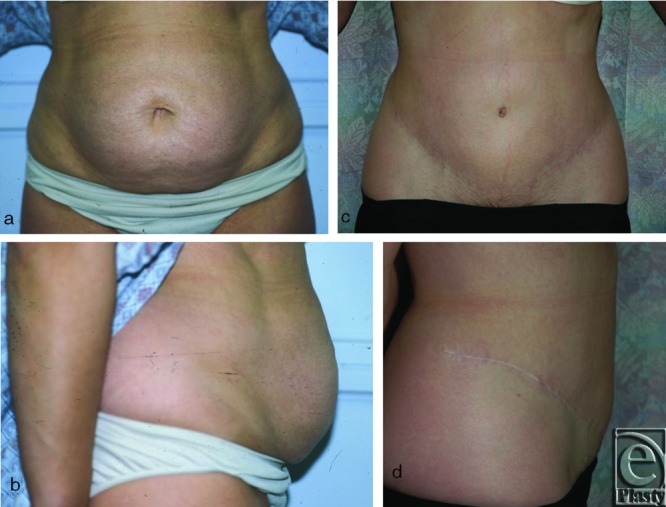
Preoperative (a, b) and postoperative (c, d) imaging of a representative patient undergoing abdominoplasty with tumescent technique.

None of the patients who were discharged the same day reported any problem with pain control postoperatively, and all patients had uneventful recovery with good postoperative wound healing in most of the patients (Fig 1). The most common complications seen were ones that involved the surgical wounds (Table 3). There were no documented deep venous thromboses, PE, hospital readmissions, major or minor skin flap necrosis, or hematomas (not associated with seromas). Wound complications occurred in 14 patients (21.6%), of which 12 had seromas (18.5%) and 2 had wound infections (3.1%). Of the patients who developed seromas, 2 were inpatients and 10 were outpatients. The 2 patients with wound infections were both outpatients. All seromas were treated with repeat clinic visits involving repeated aspirations or replacement of the flat Jackson-Pratt drain. The wound infections were treated with outpatient courses of antibiotics. A total of 7 patients required reoperations: 5 for scar revision and 2 for seroma cavity excision.

## DISCUSSION

The ability to perform abdominoplasty as an outpatient procedure decreases the procedure cost and improves patient satisfaction.^31^^-^35 It also offsets the strain on health care budget without affecting the outcomes of the procedure. One of the key features in being able to discharge the patient after an outpatient surgery is adequate postoperative pain control. A patient cannot be discharged home until he or she can demonstrate adequate pain relief on taking oral medications alone. To facilitate faster postoperative pain relief, pain pumps have been described in the literature.^23^^-^26 Use of pain pumps significantly reduces the postoperative pain and decreases the need for narcotics.^23^^,^^24^ In a study by Patel et al,^24^ 59% of patients could be discharged the same day with the use of continuous local anesthetic infusion pumps as compared with traditional inpatient abdominoplasty. Duration of hospital stay was 1.3 days shorter for the continuous infusion pump group, which translated as an average savings of about US $845 per patient. In our experience, 77% of the patients could be discharged the same day with the use of tumescence. This would be more cost-effective and would also set off the additional cost of the pain pump. Also, the patient needs to be trained about the usage of pain pumps, and there is always a risk of catheter falling out. The presence of a foreign body also predisposes the patient to pump site infection. In addition, it can be discomforting to the patient to move around with a pain pump. There has been some concern of the association of seroma with continuous infusion pain pump, but it has not been proven in the literature.^25^^,^^26^

Use of the tumescent technique reduces blood loss, bruising, and postoperative pain.^36^^,^^37^ In our series, 95% of patients could be discharged the same day with stand-alone tumescent abdominoplasty whereas 71% of the patients who underwent concurrent procedures with abdominoplasty could go home the same day. There have been few other studies evaluating the outcomes of patients with tumescent abdominoplasty.^27^^-^30 In a series of 6 patients, Abramson^27^ reported that tumescent abdominoplasty enables them to return home the same day of surgery, to mobilize sooner, and to minimize narcotic use. Abramson^27^ and Nguyen et al^28^ reported that abdominoplasty can be easily performed under conscious sedation while using the tumescent technique. Another study by Failey et al^29^ demonstrated that patients who received bupivacaine for tumescent abdominoplasty spent significantly less time in the hospital postoperatively than those who received standard lidocaine or normal saline as wetting solution. The findings of our study corroborated with the limited number of published studies using tumescent abdominoplasty. Most of the patients, with other concurrent procedures, could be discharged the same day, and all the patients reported very good postoperative pain control. We used general anesthesia for our patient series because almost 75% of our patients underwent concomitant procedures along with the abdominoplasty. On the basis of the literature review, it can be argued that these patients can be considered for conscious sedation, which would prevent the risk of general anesthesia.^30^

The overall complication rates using standard abdominoplasties with drains have been described between 18% and 54%.^12^^-^19 The most dreaded systemic complications of abdominoplasty are DVT and PE.^19^^,^^20^ In 1975, Grazer and Goldwyn^19^ reported a DVT incidence of 1.1% and a PE incidence of 0.8%. A more recent study by van Uchelen et al^20^ reported a 1.4% incidence rate of both DVT and PE. We did not have any episode of DVT and PE in our patients. One of the important considerations in all these previous studies is that abdominoplasty was performed in an inpatient setup. The patients have improved mobility when the surgery is performed in an outpatient setup, and this can explain the absence of DVT or PE in our patients.

The most common complication in our series was seroma formation (18.4%), which is within the complication rates described in the literature (1%–38%).^38^^-^41 All the seromas were initially treated with repeated aspirations or replacement of the flat Jackson-Pratt drains. We were able to treat 10 of 12 patients with seromas with this technique. However, the remaining 2 patients need excision of the seroma cavity for definitive treatment. We advocate removing excess tissue from the abdominal wall flap to not only produce a better cosmetic result but also provide added tension to help milk the lymphatics, which could decrease the seroma formation. A large number of modifications have been described in the literature to prevent the seroma formation. One of the studies compared the seroma formation in 3 groups of patients undergoing abdominoplasty with suction drains alone, with a quilting suture between the subcutaneous tissue of the flap and musculoaponeurotic layer of the anterior abdominal wall, or with fibrin sealant.^42^ On day 15, seroma formation was significantly higher in the fibrin sealant group than in the group of patients with suction drain alone or quilting stitches. However, there was no statistical difference between the 3 groups on day 30.^41^ Pollock and Pollock^43^ championed the usage of progressive tension sutures and demonstrated a seroma rate of 0.1% in 597 patients undergoing abdominoplasty. Progressive tension sutures not only helped in reducing the dead space but also immobilized the flap, which is the primary mechanism by which fluid accumulation is minimized. Another study compared the classic full abdominoplasty with a similar type of abdominoplasty except for the preservation of the Scarpa fascia and the deep fat compartment in the infraumbilical area.^17^ The Scarpa fascia preservation group had a highly significant reduction of 65.5% on the total drain output, 3 days on the time to drain removal, and 86.7% on the seroma rate. A possible explanation could be preservation of the connective tissue, lymphatic vessels, arteries, and veins of the deep fat compartment along with the Scarpa fascia. Assuming that the hypogastric area drains into the inguinal area, the preservation of lymphatics will have a lower potential to interfere with the lymph drainage of the abdominal wall.

We also recommend sharp dissection with scalpel and knife to raise the abdominal dissection as compared with electrocautery. In a recent study published by Valença-Filipe et al,^44^ abdominal dissection with scalpel had a beneficial effect on patient recovery by decreasing total drain output and days needed for drain removal as well as decreased incidence of seroma and wound healing problems when compared with abdominal dissection using electrocautery for abdominoplasty.

The wound infection rate in our series was 3%, which is comparable with that of other studies in the literature, and was effectively treated with antibiotics (progressive tension sutures). Although we close the flap under tension for better cosmesis, which can comprise blood supply to the abdominal flap, we had no incidence of minor or major skin flap necrosis. Use of tumescence also allows for meticulous hemostasis as evidenced by no hematoma in our patient group.

## CONCLUSIONS

The safety and efficiency of outpatient abdominoplasty can be further facilitated by utilizing tumescence. There were no reports of DVT or PE using this technique, and other local complications such as seroma formation were comparable with those reported in other studies. Tumescence allows the patients to be discharged the same day postoperatively, mobilize sooner, and rely less on oral narcotics at home.

## Figures and Tables

**Table 1:** Demographics of the patients undergoing abdominoplasty with tumescence (N = 65)

Demographics	*n* (%)
Sex	
Female	61/65 (93.8)
Male	4/65 (6.2)
Average age, y	45.2
Average body mass index	29.2
Chronic disease	2/65 (3.1)
Diabetes	0/65
Asthma	2/65
Previous surgical procedures	50/65 (70.9)
Cesarean delivery	21/65 (32.3)
Other gynecological surgical procedures	14/65 (21.5)
Abdominal surgical procedures	12/65 (18.5)
Prior abdominoplasty	3/65 (4.6)

**Table 2:** Distribution of the number of procedures in our study population and its effect on same-day discharge (N = 65)

Abdominoplasty + additional procedures	Alone	1	2	3	4	5
Number (%) of patients	17/65 (26)	22/65 (34)	19/65 (29)	6/65 (9)	0/65 (0)	1/65 (2)
Same-day discharge, *n* (%)	16/17 (94)	34/48 (71)				

**Table 3:** Complications from abdominoplasty and their treatment (N = 65)

Complications	*n* (%)	Treatment
Complication rate	14/65 (21.5)	
Seroma	12/65 (18.5)	Repeated aspirations/Jackson-Pratt drain—10/12
Inpatient	2/12	Seroma cavity excision—2/12
Outpatient	10/12	
Wound infection	2/65 (3)	Outpatient antibiotics (2/2)
	0/65 (0)	
	2/65 (3)	
Reoperation	7/65 (10.7)	
Scar revision	5/65 (7.7)	
Seroma excision	2/65 (3)	
